# Hyperbolic polariton-coupled emission optical microscopy

**DOI:** 10.1515/nanoph-2024-0617

**Published:** 2025-01-28

**Authors:** Shilong Li, Zhaowei Liu, Yeon Ui Lee

**Affiliations:** Interdisciplinary Center for Quantum Information, State Key Laboratory of Modern Optical Instrumentation, College of Information Science and Electronic Engineering, Zhejiang University, Hangzhou, China; Department of Electrical and Computer Engineering, University of California, San Diego, USA; Department of Physics, 34933 Chungbuk National University, Cheongju, South Korea

**Keywords:** hyperbolic polariton-coupled emission, optical imaging, directional fluorescence

## Abstract

A new type of optical microscopy based on hyperbolic polariton-coupled emission (HPCE) is demonstrated. By employing hyperbolic metamaterials as the substrate, we show a nearly 6-fold increase in fluorescence intensity in the HPCE microscope compared to total internal reflection fluorescence (TIRF) on glass substrates. Moreover, we achieve precise, time-dependent control of the fluorescence intensity by modulating the incidence angle with a galvo scanner. This tunability offers extensive potential for applications in super-resolution fluorescence microscopy and high-sensitivity sensing, enabling real-time fluorescence intensity adjustment.

## 1 Introduction

Total internal reflection fluorescence (TIRF) microscopy has become a central tool in bioimaging for studying processes near the cell-substrate interface. By introducing light at an angle exceeding the critical angle, TIRF generates an evanescent field that selectively excites fluorophores within a thin layer (approximately 100–200 nm) near the interface, significantly reducing background noise and thereby improving the signal clarity [1], [2]. This selective excitation ability enables an exceptional signal-to-noise ratio, making TIRF invaluable for studying cellular events such as vesicle fusion, membrane trafficking, and other surface-proximal dynamics. However, the achievable fluorescence enhancement is inherently limited due to the use of a glass substrate, which may restrict sensitivity in detecting subtle molecular events and weak fluorescent signals [3], [4], [5], [6].

Recent advances in engineered optical materials have positioned hyperbolic metamaterials (HMMs) as an innovative alternative substrate for enhancing fluorescence-based techniques. Owing to their hyperbolic dispersion, HMMs support high-momentum (high-*k*) states that enhance light–matter interactions at the nanoscale [7], [8], [9]. In periodic multilayer HMMs, strong coupling between surface plasmon polaritons in adjacent metal layers gives rise to bulk propagating high-*k* waves; these waves are physically understood as hyperbolic polaritons [10]. Prior theoretical and experimental studies have demonstrated that such hyperbolic polaritons in HMMs can be leveraged to achieve superior optical field confinement and signal enhancement [11], [12], [13], [14], [15]. This property allows for substantial fluorescence signal amplification compared to conventional glass substrates. Furthermore, HMMs facilitate precise control of the fluorescence signal by enabling dynamic modulation of the incidence angle, a feature that can be seamlessly integrated with conventional TIRF setups at their back focal plane, ensuring accurate adjustment of the incidence angle.

In this study, we demonstrated an optical microscope based on hyperbolic polariton-coupled emission (HPCE) by utilizing HMMs as the substrate – in place of traditional glass – with a TIRF microscope. Real-time modulation of the fluorescence intensity was achieved by adjusting the incidence angle using a galvo scanner. Fluorescence intensity was enhanced up to nearly 6-fold compared to that obtained with a glass-based TIRF. These findings highlight HPCE as a powerful tool for bioimaging, which provides enhanced sensitivity and temporal modulation of fluorescence. This work not only expands the potential applications of TIRF-based techniques but also offers new capabilities for high-resolution imaging and molecular detection where controlled fluorescence tuning is essential [16], [17], [18].

### 1.1 Experimental setup

The HPCE microscope is designed to leverage the unique dispersion of HMMs to tailor the fluorescence properties of a fluorophore. The HMMs used in the HPCE microscope are composed of a multilayer structure consisting of three pairs of alternating 10-nm silver (Ag) and 4-nm silicon dioxide (SiO_2_) layers (similar to the configuration described in previous research [9]), which facilitates high spatial frequency wave propagation with enhanced light–matter interaction.

**Figure 1:**
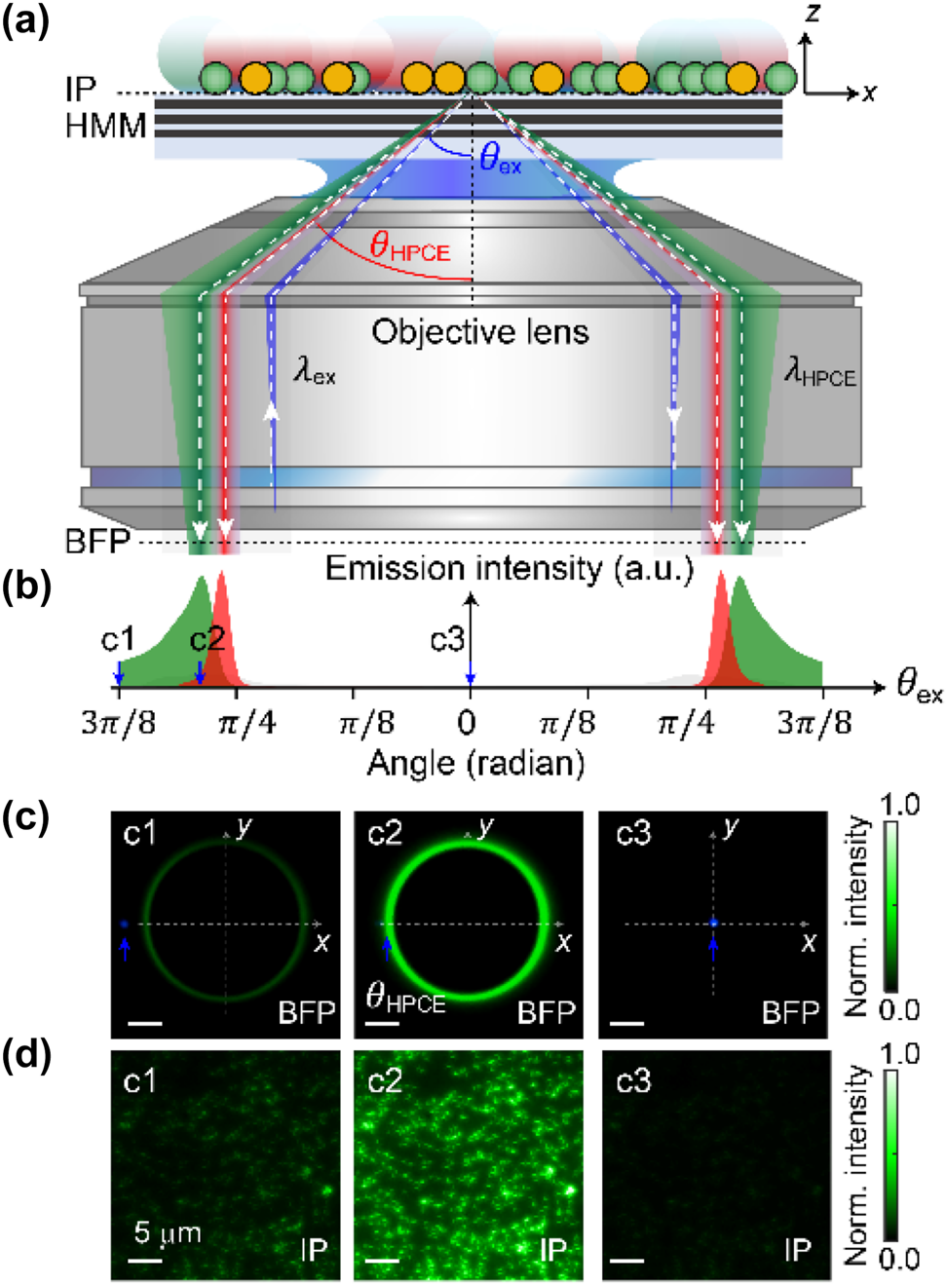
Experimental setup and imaging results of hyperbolic polariton-coupled emission (HPCE). (a) Schematic of the setup. The fluorophores, i.e., green emission fluorescent beads and red emission quantum dots, positioned on an HMM substrate (at the image plane, IP), are illuminated by a blue excitation laser (*λ*_ex_ = 488 nm) off-centered at the back focal plane (BFP) to control the incidence angle (*θ*_ex_). The emission intensity is maximized at the specific incidence angle *θ*_ex_ = *θ*_HPCE_ for the HPCE wavelength *λ*_HPCE_. (b) Wavelength-dependent angular emission profiles. The *θ*_HPCE_ values at the green and red wavelengths differ, as shown by the peaks in their respective intensity distributions, highlighting the role of hyperbolic dispersion of the HMM substrate in controlling the *θ*_HPCE_. (c) BFP images captured at different incidence angles c1, c2, and c3, demonstrating the optimal angle c2 with the highest fluorescence intensity. (d) Corresponding fluorescence images at the IP. These images also show enhanced fluorescence intensity under the optimal excitation condition (i.e., c2), illustrating the effectiveness of HPCE in improving signal sensitivity and contrast.

As shown in Figure 1a, the HPCE microscope is in an inverted microscope configuration with an HMM substrate positioned under the sample. The sample is illuminated off-centered at the back focal plane (BFP) to control the incidence angle *θ*_ex_, which allows for precise control of the excitation conditions – crucial for achieving time-dependent modulation of fluorescence intensity. The results for the fluorescence intensity as a function of *θ*_ex_ are depicted in Figure 1b. It shows that, by modulating *θ*_ex_ through a galvo scanner, one can dynamically adjust the excitation conditions, achieving a maximum enhancement in fluorescence intensity at the HPCE angle *θ*_HPCE_. Such tunability offers significant advantages in applications requiring time-resolved intensity modulation. Moreover, this tunability is wavelength-dependent due to the dispersive nature of the HMM substrate, which enables selective excitation of fluorescence at different angles, providing additional degrees of freedom for modulating the fluorescence intensity. In Figure 1c, back focal plane (BFP) images captured at different incidence angles, i.e., c1 (3*π*/8 radian), c2 (7*π*/32 radian), and c3 (0 radian), demonstrate the optimal angle c2 with the highest fluorescence intensity. The corresponding fluorescence images (Figure 1d) at the image plane (IP) show enhanced fluorescence intensity under the optimal excitation condition (i.e., c2), illustrating the effectiveness of HPCE in improving signal sensitivity and contrast.

## 2 Experimental results

### 2.1 Comparison of TIRF and HPCE

**Figure 2:**
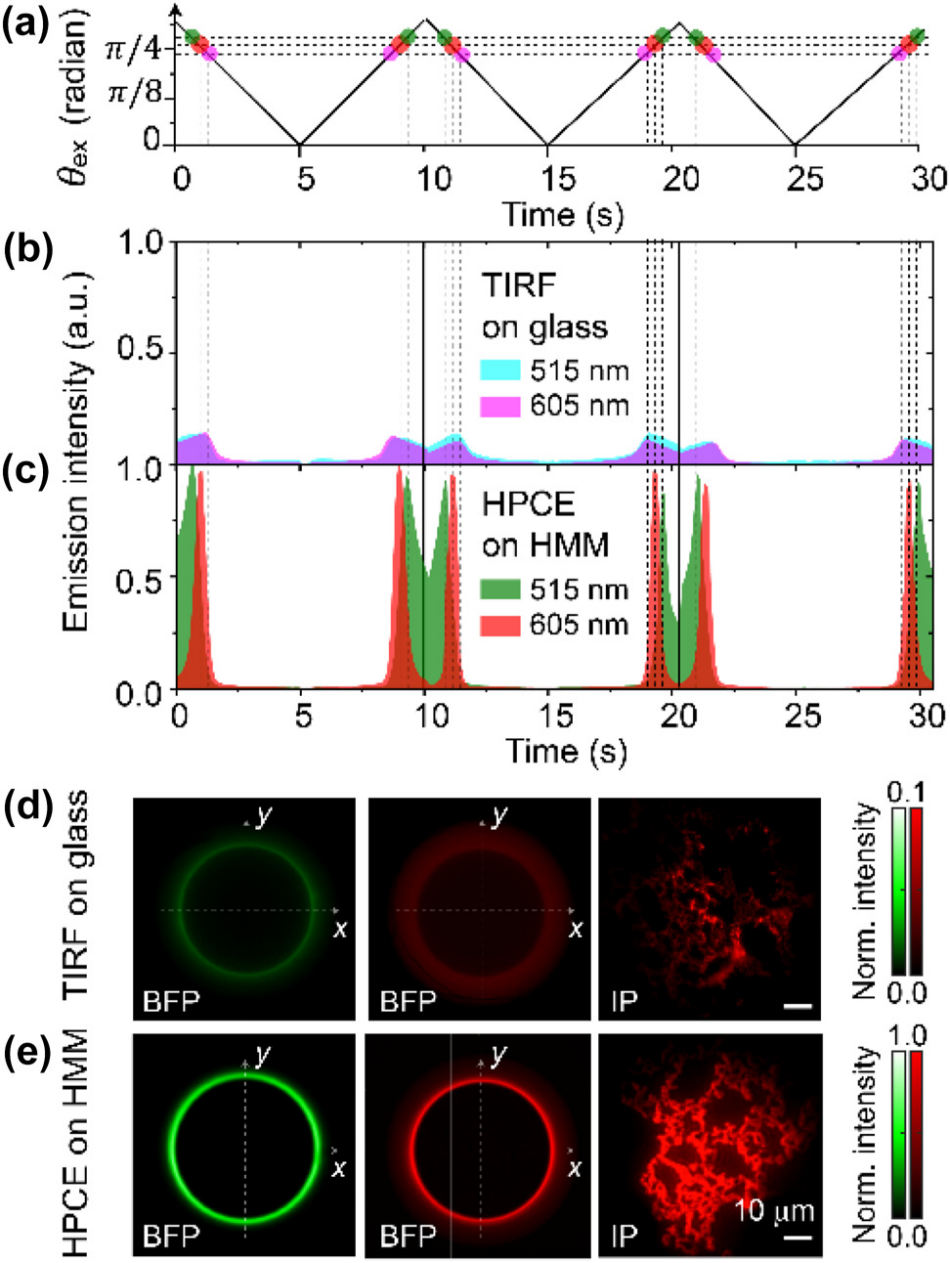
Comparison of fluorescence emission for red quantum dots (605 nm) and green fluorescent beads (515 nm) under TIRF using glass and HPCE using HMM. (a–c) Incidence angle modulation over time (a) and the resultant emission intensity variations for TIRF on glass (b) and HPCE on HMM (c), respectively. (d, e) Corresponding BFP and IP images for TIRF on glass (d) and HPCE on HMM, respectively.

The time-dependent modulation of the incidence angle used in the experiment is shown in Figure 2a. The angle sweeps between 0 and 3*π*/8 radians and is adjusted to enhance fluorescence emission. Figure 2b shows the resultant emission intensity variation under TIRF using glass. Even though TIRF gives rise to enhanced emission intensity at the total internal reflection angle of the air/glass interface, the emission intensity remains relatively low and lacks distinct peaks due to the limited enhancement capability of TIRF when using glass. On the other hand, the fluorescence emission for HPCE on an HMM substrate (Figure 2c) exhibits strong and clear intensity peaks for both green and red wavelengths at specific incidence angles, demonstrating the emission engineering ability of the HPCE.

The BFP and IP images of both green fluorescent beads and red quantum dots under TIRF using glass (Figure 2d) and HPCE using HMM (Figure 2e) are shown. For TIRF on glass, the fluorescence emission is weak and lacks clear angular control, resulting in lower signal intensity and contrast in the IP images. In contrast, for HPCE on HMM, BFP images of both green fluorescent beads and red quantum dots display distinct ring patterns, indicating strong, angle-selective enhancement of these fluorophores. Moreover, the corresponding IP images exhibit significantly increased fluorescence intensity for both the green and red fluorophores, confirming that HPCE on HMM provides superior signal enhancement and contrast compared with TIRF on glass.

### 2.2 Analysis of HPCE physical origin

**Figure 3:**
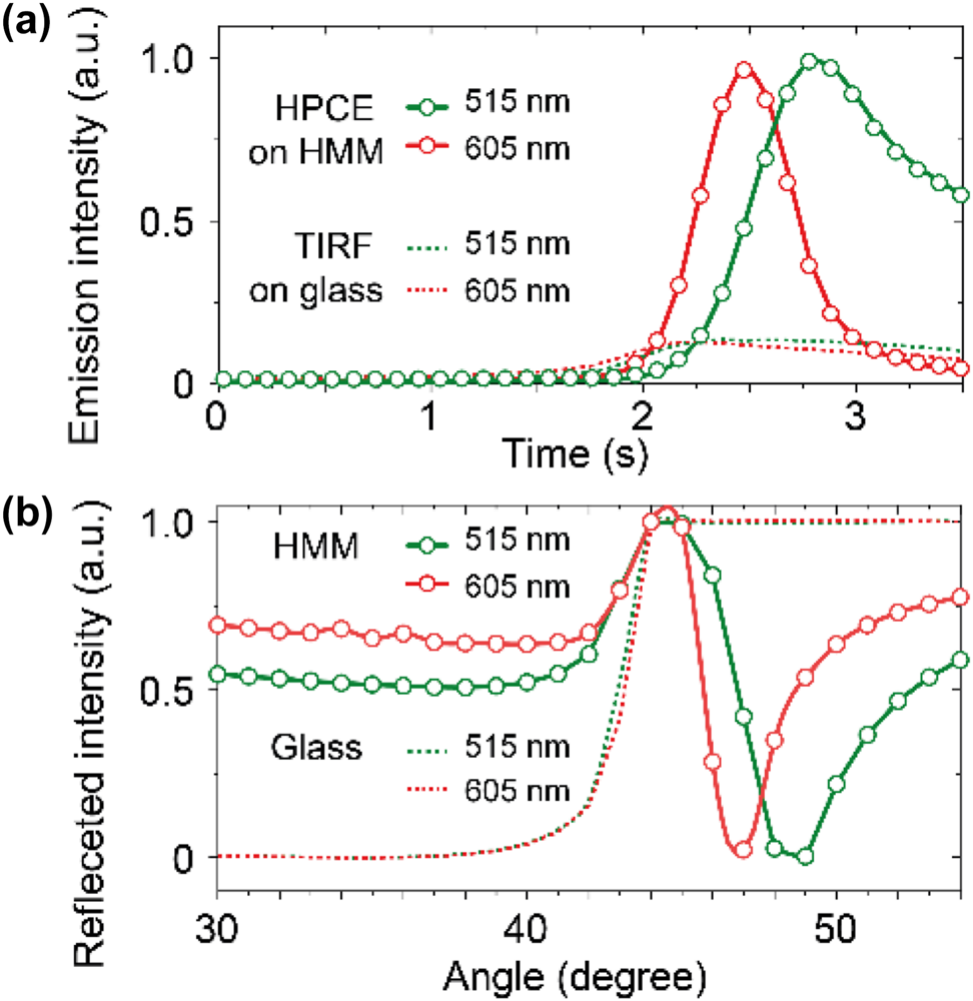
Analysis of the physical origin behind the HPCE microscope. (a) Angle-dependent emission results. Emission intensity modulation is achieved by varying the incidence angle over time for both the green (515 nm) and red (605 nm) wavelengths under two conditions, i.e., HPCE using an HMM substrate and TIRF using a glass substrate. (b) Corresponding attenuated total internal reflection (ATR) results. Reflection dips reach their minimum at the resonance angles where hyperbolic polariton excitation occurs, aligning with the peaks observed in HPCE emission intensity.

The physical origin of HPCE is the excitation of hyperbolic polaritons supported by the HMMs used, which has been confirmed through precise measurement of the angle-dependent emission and the attenuated total internal reflection (ATR) as shown in Figure 3. In the angle-dependent emission experiments, peaks in emission intensity are expected to be observed at the optimal angles for hyperbolic polariton excitation within the HMMs when strong coupling between the incident light and the hyperbolic polaritons takes place. To find out such conditions, the incidence angle was systematically varied from 30° to 55° in 1° steps, while the corresponding emission intensity was monitored. The results for both the green (515 nm) and red (605 nm) wavelengths under the two conditions, i.e., HPCE using an HMM substrate and TIRF using a glass substrate, are summarized in Figure 3a. The HPCE on HMM shows a significant increase in emission intensity for both wavelengths, with a distinct time-dependent modulation. This modulation is attributed to angle sweeps across optimal incidence angles, which align with the hyperbolic polariton excitation in the HMM, resulting in enhanced fluorescence emission. In contrast, TIRF on glass displays lower and more stable intensity modulation, indicating limited enhancement due to the lack of hyperbolic polariton excitation.

The attenuated total internal reflection (ATR) experiments [19], [20] were conducted to analyze how light interacts with the HMM substrate across this range of incidence angles (Figure 3b). The ATR results reveal the angle-dependent excitation characteristics of the hyperbolic polaritons, providing insight into the resonance conditions that enhance HPCE. As the incidence angle is swept from 30° to 55°, the HMM substrate exhibits pronounced dips in reflected intensity around specific angles for each wavelength. The reflection dips observed in the ATR experiment align with the emission intensity peaks, confirming the role of hyperbolic polariton excitation in achieving enhanced fluorescence in HPCE. In contrast, the ATR experiment with the glass substrate shows no significant changes in reflected intensity, underscoring the lack of angle-dependent coupling effects.

### 2.3 Simulations of optical field enhancement in HPCE

**Figure 4:**
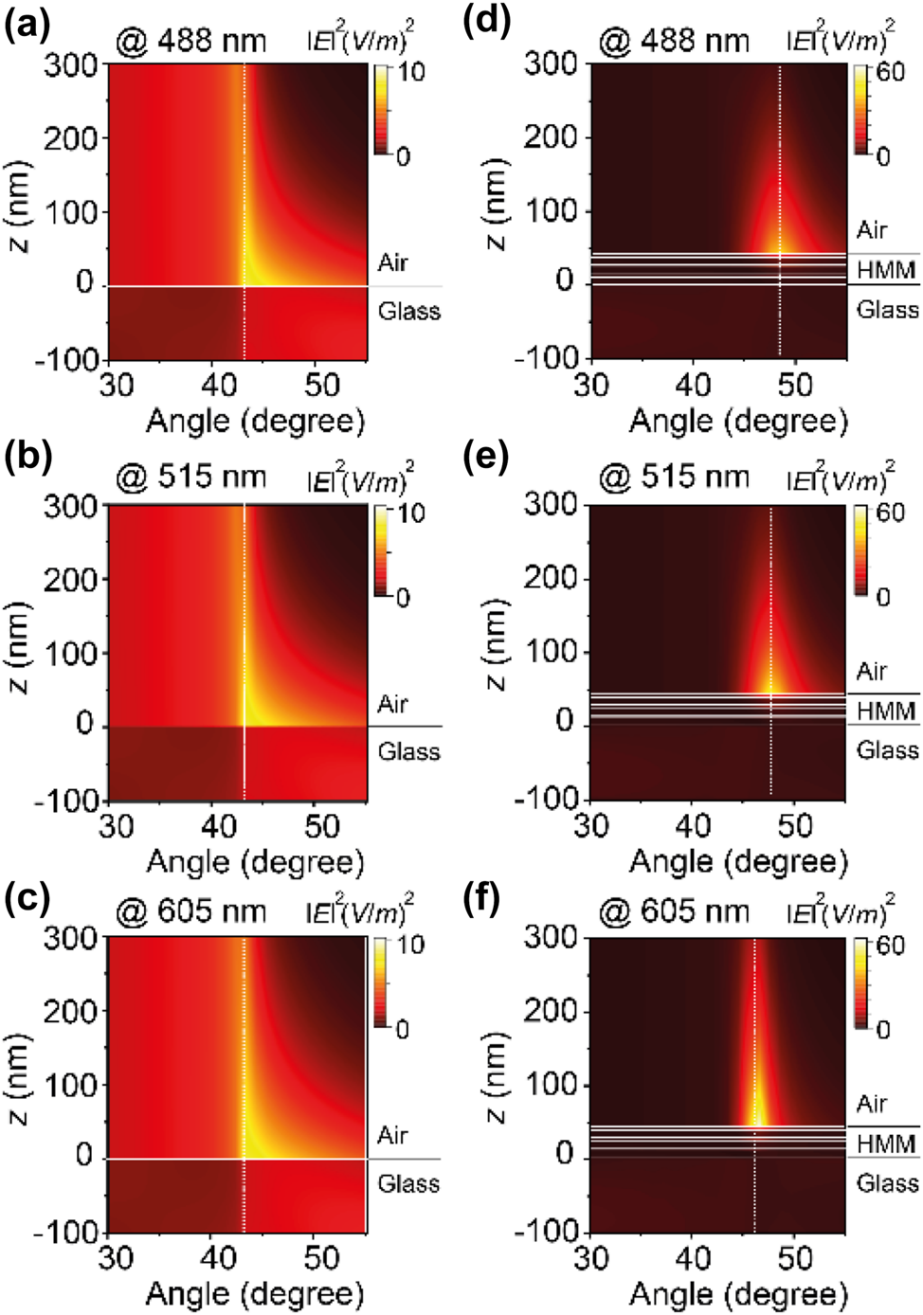
Analysis of the optical field enhancement in the HPCE microscope. (a–f) FDTD simulation results showing field enhancement using both the glass (a–c) and HMM (d–f) substrates at wavelengths of 488 nm, 515 nm, and 605 nm, respectively, for each substrate.

To have a better understanding of the enhanced fluorescence in the HPCE microscope, numerical simulations were carried out based on the finite difference time domain (FDTD) method. Figure 4 summarizes the simulation results of the excitation field enhancement using both the glass and HMM substrates at wavelengths of 488 nm, 515 nm, and 605 nm for each substrate, respectively. In these simulations, the excitation field is modeled as an obliquely incident plane wave from the glass side. The HMM substrate used in the HPCE microscope is a multilayer structure consisting of three pairs of alternating 10-nm silver (Ag) and 4-nm silicon dioxide (SiO_2_) layers. The intrinsic dielectric permittivity values of Ag and SiO_2_, as reported in the Palik database [21], were used for each material. As shown in Figure 4a–c, the field intensity, ∣*E*∣^2^, is localized near the glass–air interface with a limited intensity peak around the critical angle, exhibiting the typical optical response of TIRF. The excitation field decays rapidly beyond the penetration depth, limiting the enhancement effect achievable on the glass substrate. Figure 4d–f shows the excitation field intensity using the HMM substrate. The incident plane wave excites surface plasmon polaritons at the interfaces of individual metal layers, which subsequently couple to form bulk hyperbolic polaritons. A significantly large field enhancement across a broad range of incidence angles is observed, owing to the hyperbolic dispersion of the HMM. This extended angular range, combined with the higher field intensity near the HMM surface, enables a more robust excitation field that is not limited to the critical angle region, as observed in TIRF. Although the radiative decay rate enhancement is not included in these simulations, the excitation field enhancement alone suggests that HPCE using an HMM substrate can achieve over six times the fluorescence emission enhancement compared to conventional TIRF using the glass substrate. This result indicates that the HMM structure, by enhancing the excitation field, also significantly improves the emission efficiency, which is consistent with experimental measurements (Figure 3a). These FDTD simulations confirm that the excitation field enhancement on HMMs is significantly greater than that on glass, even without accounting for radiative decay rate enhancements, such that HMM-based substrates inherently boost fluorescence signal through excitation field amplification. It is worth noting that the contribution of radiative decay rate enhancement is minimal in nonpatterned HMM substrates compared to nonradiative effects [22]; additionally, radiative decay rate enhancement does not depend on the incidence angle, which is crucial for HPCE-induced emission enhancement.

### 2.4 Optimization of HPCE enhancement

**Figure 5:**
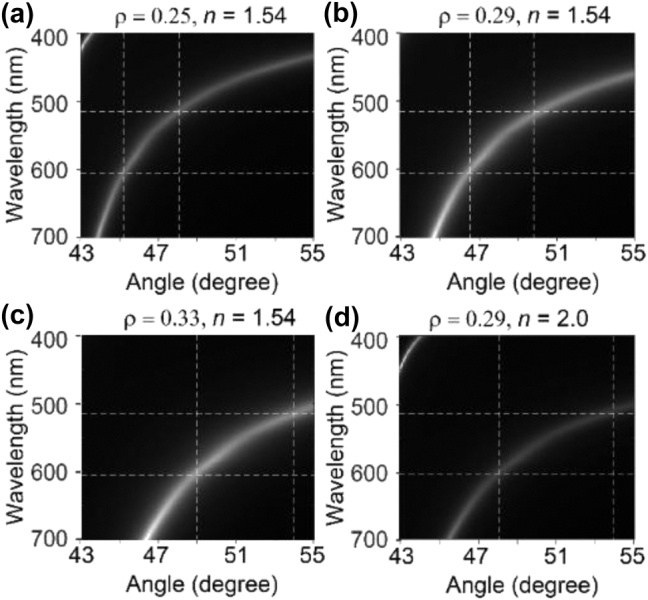
Calculated hyperbolic polariton dispersion for HMMs with varying material parameters. (a) Hyperbolic polariton dispersion with a fill fraction *ρ* = 0.25 and refractive index of the dielectric layer *n* = 1.54. (b) Dispersion for *ρ* = 0.29 and *n* = 1.54, which are the parameters used in our experiment. (c) Dispersion for *ρ* = 0.33 and *n* = 1.54. (d) Effect of increasing the dielectric refractive index to *n* = 2.0 for *ρ* = 0.29, demonstrating how material parameters influence the hyperbolic polariton excitation conditions across wavelength and incidence angle.

Hyperbolic polariton excitation in HMMs leads to significant field enhancement at the HMM surface, which in turn results in HPCE enhancement. Figure 5 shows the calculated hyperbolic polariton dispersion of HMMs composed of three pairs of alternating metal (Ag) and dielectric layers. The hyperbolic polariton modes are calculated through the transfer matrix method. The wavelength-dependent nature of hyperbolic dispersion is influenced by the material properties of the HMM, including the thickness of the metal and dielectric layers, the fill fraction $\rho =t_{\text{m}}/\left(t_{\text{m}}+t_{\text{d}}\right)$ of the metal, where *t*_m_ and *t*_d_ are the thicknesses of the metal and dielectric films, and the refractive index *n* of the dielectric material. HPCE occurs when the momentum of the excitation beam matches the momentum of the hyperbolic polariton dispersion. This matching condition depends on the hyperbolic dispersion, which varies with wavelength and is directly affected by the optical properties of the metal and dielectric layers. As a result, the optimal incidence angle for HPCE varies depending on the fluorescence wavelengths of the sample. By tuning the material parameters – such as layer thicknesses and refractive indices – and adjusting the incidence angle of the excitation beam, HPCE can be optimized for specific fluorescence wavelengths to achieve maximum fluorescence enhancement. The choice of materials and their structural parameters plays a critical role in determining the hyperbolic dispersion and the conditions for optimal HPCE. This enables the customization of fluorescence enhancement for imaging applications by aligning the HPCE conditions with the spectral properties of the fluorescent sample.

## 3 Conclusion and discussion

In conclusion, we have demonstrated an HPCE microscope using an HMM substrate with a significant fluorescence enhancement compared to the conventional TIRF microscope on glass. The nearly 6-fold improvement in emission intensity underscores the effectiveness of the HMM in enhancing excitation fields. The angle-dependent control over fluorescence intensity enabled by the hyperbolic dispersion of the HMM allows for selective and tunable excitation, which is beneficial for high-sensitivity fluorescence imaging applications. Such dynamic modulation capability is promising for advanced applications requiring high sensitivity and controlled fluorescence emission including live-cell imaging, biomolecular detection, and nanoscale optical sensing [23], [24], [25]. In addition, the hyperbolic dispersion of the HMM substrate enables high-momentum optical modes that amplify the excitation field near the surface, facilitating stronger light–matter interactions. This dispersion characteristic allows for efficient coupling at specific incidence angles, where hyperbolic polariton excitation occurs, thereby maximizing fluorescence emission. Additional work is needed to explore the influence of different HMM designs and materials on the HPCE performance. For instance, varying the thickness or materials in the HMM layers could provide insights into optimizing fluorescence for specific applications (Figure 5). The anisotropic nature of HMMs plays a crucial role in the angular dependence of emission observed in our study. Hyperbolic dispersion in HMMs enables the coupling of fluorescence emission into high-momentum modes (hyperbolic polaritons), which are not accessible in isotropic materials such as glass. This angular dependence arises because the hyperbolic polariton modes depend on the direction of the emission relative to the optical axis and the momentum-matching condition. As a result, the emission intensity exhibits a strong dependence on both the emission angle and the excitation beam incidence angle under HPCE conditions, and this is not observed in conventional TIRF on glass substrates.

While the anisotropic nature of HMMs inherently influences the angular dependence of emission through hyperbolic dispersion, deviations from theoretical predictions may arise due to surface roughness, material defects, and fabrication inaccuracies. For example, surface roughness or defects can scatter the hyperbolic polariton modes. This scattering reduces the efficiency of polariton coupling and thus results in lower fluorescence enhancement than predicted by the theoretical model. Additionally, defects may introduce nonuniformities in the excitation field, further affecting the observed angular dependence. Small variations in layer thicknesses during fabrication can shift the hyperbolic polariton dispersion, which alters the optimal incidence angles for HPCE and causes discrepancies between the model and experimental results.

The ability to dynamically modulate fluorescence intensity by adjusting the incidence angle with a galvo scanner provides a novel tool for applications that require temporal control of emission. The integration of HMM substrates with fluorescence microscopy enhances fluorescence intensity and enables tunable emission. For example, it could be particularly valuable in super-resolution microscopy, such as structured illumination microscopy (SIM) and single-molecule localization microscopy (SMLM), which rely on signal reconstruction. Combining HPCE with SIM allows the high-momentum hyperbolic polaritons to serve as structured illumination, enabling reconstructed image resolutions to improve by more than an order of magnitude [9], [26]. Additionally, the HPCE effect provides improved sectioning capabilities, leading to images with higher contrast and sharpness. In SMLM, HPCE can enhance the signal-to-noise ratio (SNR), which is critical for enhancing localization precision. The increased SNR makes it easier to achieve subdiffraction resolution, as the precision of molecule localization depends heavily on fluorescence signal strength. Integrating HPCE with other imaging modalities, such as confocal or multiphoton microscopy, could further extend its applicability. By combining the techniques with HMM-based substrates, it may be possible to achieve new levels of sensitivity and specificity in fluorescence imaging.

A key advantage of HPCE lies in its ability to extend the photobleaching lifetime of fluorophores through hyperbolic polariton excitation [22], [27]. This addresses one of the primary challenges in fluorescence microscopy – photodamage and rapid photobleaching – making HPCE an effective strategy for overcoming these limitations in high-intensity imaging applications.
